# High Hydrostatic Pressure Processing of Human Milk Increases Apelin and GLP-1 Contents to Modulate Gut Contraction and Glucose Metabolism in Mice Compared to Holder Pasteurization

**DOI:** 10.3390/nu14010219

**Published:** 2022-01-05

**Authors:** Eve Wemelle, Lucie Marousez, Marie de Lamballerie, Claude Knauf, Jean Lesage

**Affiliations:** 1 INSERM U1220, Institut de Recherche en Santé Digestive (IRSD), Université Paul Sabatier, Toulouse III, CHU Purpan, Place du Docteur Baylac, CS 60039, CEDEX 3, 31024 Toulouse, France; eve.wemelle@inserm.fr; 2 European Associated Laboratory (EAL) «NeuroMicrobiota», International Research Projects (IRP) INSERM, 1000 Brussels, Belgium; 3 European Associated Laboratory (EAL) «NeuroMicrobiota», International Research Projects (IRP) INSERM, 31024 Toulouse, France; 4 Univ. Lille, Inserm, CHU Lille, U1286-INFINITE-Institute for Translational Research in Inflammation, F-59000 Lille, France; lucie.marousez@univ-lille.fr; 5 UMR CNRS 6144 GEPEA, ONIRIS, CS 82225, 44322 Nantes, France; marie.de-lamballerie@oniris-nantes.fr

**Keywords:** human milk, high hydrostatic pressure, holder pasteurization, GLP-1, apelin, glucose metabolism

## Abstract

Background: High hydrostatic pressure (HHP) processing is a non-thermal method proposed as an alternative to Holder pasteurization (HoP) for the sterilization of human breast milk (BM). HHP preserves numerous milk bioactive factors that are degraded by HoP, but no data are available for milk apelin and glucagon-like peptide 1 (GLP-1), two hormones implicated in the control of glucose metabolism directly and via the gut–brain axis. This study aims to determine the effects of HoP and HHP processing on apelin and GLP-1 concentrations in BM and to test the effect of oral treatments with HoP- and HHP-BM on intestinal contractions and glucose metabolism in adult mice. Methods: Mice were treated by daily oral gavages with HoP- or HHP-BM during one week before intestinal contractions, and glucose tolerance was assessed. mRNA expression of enteric neuronal enzymes known to control intestinal contraction was measured. Results: HoP-BM displayed a reduced concentration of apelin and GLP-1, whereas HHP processing preserved these hormones close to their initial levels in raw milk. Chronic HHP-BM administration to mice increased ileal mRNA nNos expression level leading to a decrease in gut contraction associated with improved glucose tolerance. Conclusion: In comparison to HoP, HPP processing of BM preserves both apelin and GLP-1 and improves glucose tolerance by acting on gut contractions. This study reinforces previous findings demonstrating that HHP processing provides BM with a higher biological value than BM treated by HoP.

## 1. Introduction

Human breast milk (BM) donated to human milk banks (HMBs) is the sole diet for preterm infants when maternal BM is not available. Pasteurization of donated BM is an essential step to inactivate pathogens to ensure microbial safety for preterm babies as, due to immature gut, these infants are at high risk of developing diseases such as necrotizing enterocolitis and sepsis [1]. The most common method used in HMBs worldwide to treat BM is the Holder pasteurization (HoP) which consists of heating BM to 62.5 °C for 30 min [2]. However, owing to this heating, HoP degrades in part numerous milk heat-sensitive bioactive factors, including hormones [2]. High hydrostatic pressure (HHP) processing is a non-thermal method recently proposed as an alternative to HoP for BM sterilization [3]. Several studies have shown that HHP preserves numerous sensitive bioactive factors that are degraded by HoP [3] but, few data are available for milk hormones, and no in vivo studies have been performed so far.

More than twenty peptide hormones have been characterized in BM [4,5]. It was demonstrated that numerous of these hormones are implicated in gut maturation as well as in energy homeostasis for the newborn [4,5]. Among them, apelin and glucagon-like peptide-1 (GLP-1) were proposed to control glucose metabolism by modulating insulin release and sensitivity directly but also by targeting the gut–brain axis, which controls glucose homeostasis [5,6]. For example, we demonstrated in mice that oral apelin administration controls intestinal smooth muscle cells contractions and communicate through the enteric nervous system (ENS) with the hypothalamus to control glycemia [6]. Such compounds such as apelin that act on the ENS to control glucose utilization via the brain is named enterosynes [7]. GLP-1 is also an important regulator of glycemia. GLP-1 is a gut hormone acting on the pancreas and known as an incretin [8]. GLP-1 also has extrapancreatic sites of action, such as in the portal vein [9] and in the brain [10,11], especially for the control of glucose homeostasis in response to enteric glucose sensing. Although it remains to demonstrate that these two hormones exert in the newborn the same metabolic roles as in adults, we postulate that changes in BM apelin and GLP-1 levels may lead to altered glucose homeostasis in newborns.

Here, we investigate the effect of HoP- and HHP-treatments of BM on the concentration of apelin and GLP-1 and test in vivo the effect of chronic (7 days) oral administration of HoP- and HHP-BM on intestinal contractions and glucose metabolism in adult mice.

## 2. Materials and Methods

### 2.1. Milk Collection and HoP and HHP Processing

Frozen BM samples from 11 donors were provided by the regional HMB (Lactarium Régional de Lille, Jeanne de Flandre Children’s Hospital, CHU Lille). Donors provided written, informed consent for the use of their milk for this research purpose. After thawing of milk samples, 8 different batches of BM were created under sterile conditions by mixing various volumes (from 10 to 30 mL) of all BM samples, primarily in order to homogenize BM composition between batches. Three aliquots of BM were prepared for each batch: one fraction was stored at −80 °C without any other treatment (raw milk sample (RM)); one fraction was subjected to HoP according to the standard pasteurization protocol (62.5 °C for 30 min) in our regional HMB; the last fraction was subjected to HHP processing as previously described [12]. Briefly, the set of HHP parameters was as follows: pressure = 350 MPa, temperature = 38 °C, VA (application rate) = 1 MPa.s^−1^, number of cycles = 4 cycles, duration of each cycle = 5 min and a latency time with normal pressure between each cycle of 5 min. Samples were stored at −80 °C until analysis.

### 2.2. Quantification of Apelin and GLP-1 in Milk Samples

Apelin and GLP-1 were quantified using commercial ELISA kits (GLP-1 elisa: HUFI00805, ELISA Genie, Dublin, Ireland; apelin-12 elisa: EKE-057-23, Phoenix Pharmaceuticals, Strasbourg, France) in raw milk (RM), HoP- and HHP-BM samples using whole milk.

### 2.3. Mice

Nine-week-old male C57BL/6J mice (Charles River Laboratory, l’Arbresle, France) were housed in controlled environment (room temperature of 23 °C ± 2 °C, 12 h’ daylight cycle). Food and water were proposed *ad libitum*. Oral gavage of HoP- or HHP-BM (100 μL) was performed daily during 7 days before experiments.

### 2.4. Oral Glucose Tolerance Test (OGTT)

After a 10 h of fasting, an OGTT (3 g of glucose/kg of body weight) was performed in mice at day 7. Glycaemia was recorded from −30 to 120 min, and insulin and glucagon levels were measured and analyzed at −30, and +15 min as previously described [13].

### 2.5. Measurement of Isotonic Intestinal Contractions

Mice were euthanized under fed conditions. Duodenum, jejunum, ileum and colon, were washed and incubated with an oxygenated Krebs-Ringer solution, pH 7.4, for 20 min at 37 °C. Intestinal fragments were attached to an isotonic transducer (MLT7006 Isotonic Transducer, Hugo Basile, Comerio, Italy). The lever is adjusted to have a load of 1 g (10 mN). Each intestinal fragment was immersed in an organ bath containing an oxygenated Krebs-Ringer solution maintained at 37 °C. After a recovery period of 15 min, 100 μL of Krebs-Ringer (vehicle) solution or BM (HoP- or HHP-BM) was put directly in the organ bath and data were collected for 15 min. After attaching the intestinal segments, basal contractions were recorded for 15 min. For acute treatments, 100 μL of Krebs-Ringer (vehicle) solution or BM (HoP- or HHP-BM) were added to the medium, and contractions were recorded for 15 min. Contraction amplitudes and frequencies are presented as percentages relative to the basal response (before the injection of vehicle or milk) [13]. For chronic treatment, mice were euthanized after 7 days of milk gavage, and basal contractions were recorded for 15 min. Contraction amplitudes are presented as amplitude mean (mN), and contraction frequencies are presented as numbers of contractions per minute [13]. All recordings were performed using Labchart 8.0 software (AD Instruments, Colorado Springs, CO, USA).

### 2.6. Gene Expression

Homogenization of tissues, total RNA extraction, reverse transcription and real-time PCR were performed as previously described in detail [13]. The sequences of primers used in this study are presented in Table 1. Quantification of gene expression was performed using the comparative Ct (threshold cycle) method, and data were normalized to HPRT expression.

### 2.7. Statistics

Results are presented as mean ± SEM. GraphPad Prism 7.0. software (San Diego, CA, USA) was used to analyse the data. Outliers were detected via a Grubb’s test. A D’Agostino-Pearson test was used to evaluate the normality of variables. Statistical differences were then tested by paired *t*-test, one-way ANOVA, or two-way ANOVA test according to sample normality assessment results. Variations were considered as significant when *p* value was <0.05.

## 3. Results

### 3.1. Apelin and GLP-1 Levels in RM-, HoP- and HHP-BM

**Figure 1:**
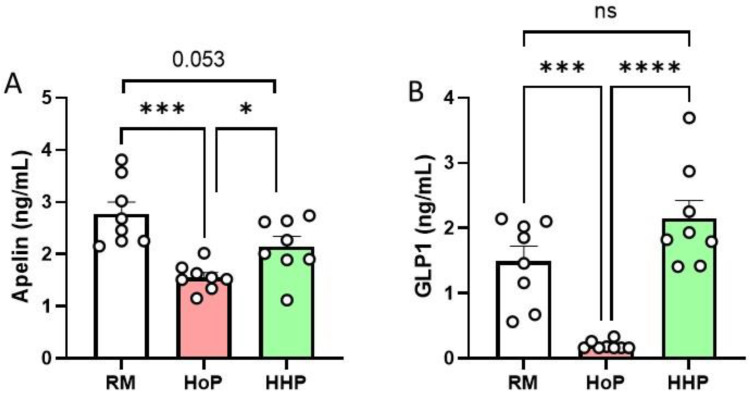
Concentrations of apelin (**A**) and GLP-1 (**B**) in raw human milk (RM) and after Holder pasteurization (HoP) and high hydrostatic pressure (HHP) processing of BM. Data are presented as mean ± SEM. Asterisks correspond to level of statistical significance: * *p* < 0.05; *** *p* < 0.001; **** *p* < 0.0001.

Compared to raw milk (RM) and HHP-BM, HoP treatment reduced apelin (−41% compared to RM, Figure 1A) and GLP-1 levels (−83% compared to RM, Figure 1B). On the opposite, no significant variation was observed for apelin (Figure 1A) and GLP-1 (Figure 1B) levels between RM- and HHP-BM.

### 3.2. Impact of HoP- and HHP-BM on Intestinal Contractions and Glucose Metabolism in Mice

**Figure 2:**
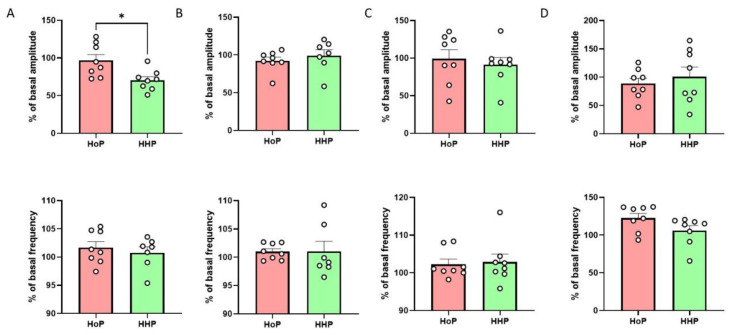
HHP-BM decreases duodenal contraction amplitude. Ex vivo measurement of duodenum (**A**), jejunum (**B**), ileum (**C**) and colon (**D**) mechanical contraction amplitude and frequency in response to HoP- or HHP-BM added in the medium (100 µL, *n* = 7–8). Results are expressed as a percentage of the basal contractions amplitude or frequency. * *p* < 0.05 compared to HoP-BM.

The addition of HHP-BM directly in the survival medium significantly decreased the amplitude of duodenum contraction compared to HoP-BM without modification of the frequency of contractions (Figure 2A). This effect was specific to the duodenum as we did not observe any significant variation in the jejunum, ileum and colon contractions between the two experimental conditions (Figure 2B–D).

**Figure 3:**
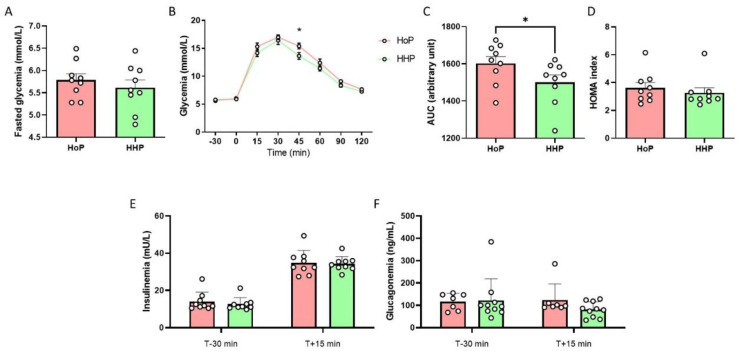
A chronic oral HHP-BM treatment improves glucose tolerance in adult mice. Oral Glucose Tolerance Test (OGTT) in fasted mice after an oral administration of HoP- or HHP-BM (100 µL/day during one week; *n* = 9 per group). (**A**) Fasted glycemia, (**B**) Glycemia during OGTT and the area under the curve (AUC) (**C**) and HOMA index (**D**), plasma insulin (**E**) and glucagon levels (**F**). * *p* < 0.05 compared to mice treated with HoP-BM.

Then, we performed experiments in response to a seven day-chronic oral gavage of mice with HHP- or HoP-BM. No significant variation was observed between mice treated with HHP- and HoP-BM concerning fasted glycemia (Figure 3A). Mice treated with HHP-BM presented an improvement of glucose tolerance (Figure 3B–D) without modification of plasma insulin and glucagon levels (Figure 3E,F).

**Figure 4:**
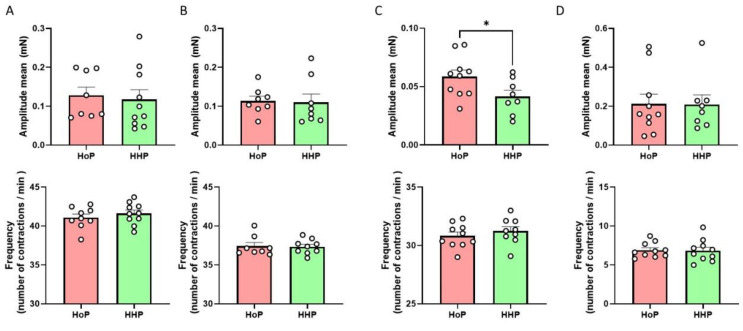

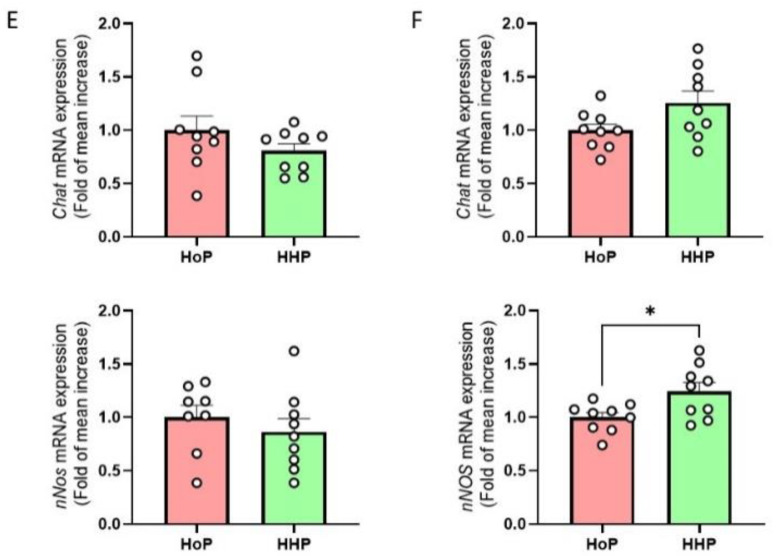
Chronic oral HHP milk treatment decreases ileal contraction amplitude. Ex vivo measurement of duodenum (**A**), jejunum (**B**), ileum (**C**) and colon (**D**) mechanical contraction amplitude and frequency in response to chronic oral treatment with HoP- or HHP-BM (100 µL/day during one week; *n* = 8–10 per group). Expression of *nNos* and *Chat* mRNAs in the duodenum (*n* = 9) (**E**) and in the ileum (**F**). * *p* < 0.05 compared to HoP-BM treated mice.

Regarding the impact of chronic (7 days) oral gavage of mice with HHP- or HoP-BM on intestinal contractions, no significant variation was observed concerning the contraction of the duodenum (Figure 4A), the jejunum (Figure 4B) and the colon (Figure 4D). On the opposite, our results showed that HHP-BM treated mice presented a significant decrease in the amplitude of contractions in the ileum without any modification of the frequency compared to mice treated with HoP-BM (Figure 4C). The HHP-BM or HoP-BM treatments of mice had no impact on the mRNA expression of nNos or Chat enzymes in the duodenum (Figure 4E), but HHP-BM treatment increased the mRNA expression of nNos, but not Chat enzyme in the ileum compared to HoP-BM treated mice (Figure 4F).

The impact of BM treatments on glucose tolerance and gut motility was not associated with variations of body weight in the different experimental groups (Before: HoP group = 27.93 ± 0.12 g, HHP group = 27.85 ± 0.10 g; After: HoP group = 28.42 ± 0.16 g, HHP group = 28.21 ± 0.10 g).

## 4. Discussion

Milk hormones are non-nutritive bioactive compounds that exert beneficial short- and long-term health effects as well as possible long-term metabolic programming in newborns, as suggested by animal studies [4]. Here, we first compared the effect of HoP, the reference method for BM sterilization in HMBs, to the new alternative method of HHP processing on the preservation of milk apelin and GLP-1. We discovered that HHP processing protects milk apelin and GLP-1 from degradation compared to HoP at concentrations similar to that observed in RM. In accordance, previous studies have shown that HoP of BM reduces others peptide hormones such as leptin, insulin and adiponectin [14,15]. Our result reinforces the fact that milk hormones are largely degraded by HoP, leading to sterilized BM with reduced hormonal benefits for preterm newborns. Thus, through the preservation of both apelin and GLP-1, HHP-treatment of BM in HMBs could improve glucose homeostasis and metabolic health in preterm infants who receive it.

In line with this hypothesis, the second objective of our study was to test in vivo the effect of a chronic gavage with HoP- and HHP-BM on intestinal contractions and glucose metabolism in adult mice. First, in a preliminary ex vivo study, we discovered that the addition of HHP-BM in the incubation medium of mouse duodenum significantly reduced the amplitude of contractions. This first rapid effect probably implies the action of milk apelin, which is now considered an enterosyne [7]. Indeed, our group has discovered the “enterosynes concept”, showing that some compounds, including apelin, could decrease the duodenal contraction intensity to restore the altered gut–brain axis activity in diabetic mice and then improve glucose tolerance [6]. The effect of apelin was observed in ex vivo conditions similar to the protocol used here but also in response to a one-week oral gavage with apelin [6]. However, in this study, we observed that HHP-BM treated mice had a decreased ileum contraction intensity and an improved glucose tolerance compared to mice treated with HoP-BM. Thus, it seems that chronic treatment with BM may rather affect gut contractions in the distal part of the small intestine, whereas the rapid effect of milk compounds in our ex vivo model mainly acts on the duodenum. Further studies are therefore needed to confirm this hypothesis between these two different models. Previous data from our team demonstrated that oral peptide (others than apelin) [16] or lipid [13] treatments in mice decrease duodenal contraction leading to the genesis of a nervous message from the intestine to the hypothalamus. By acting on the ENS, these bioactive molecules are considered enterosynes since this gut–brain axis message participates in the improvement of glucose tolerance by facilitating glucose entry in tissue such as in the adipose tissue, in the liver and/or in muscles [7]. In the ileum, GLP-1 is known to activate the “ileum brake” to inhibit small intestinal contractions [17,18] by acting on the ENS. This “ileum contraction to brain axis” could be the first mode of communication between the intestine and the brain in our experimental in vivo model. The second mode of communication could be due to the existence of GLP-1 receptors on vagal afferent sensitive neurons in the gut. Indeed, Borgmann et al. [19] have shown that the chemogenetic activation of GLP-1 receptor in the intestine reduces glucose levels. Third, GLP-1 receptors are implicated in the activation of hepatic portal vein glucose sensors leading to a decrease in glycemia via an afferent nervous message [9]. In turn, the brain can send an efferent nervous message that favors glucose entry into tissue. At this time, we can speculate that the increase in GLP-1 in HHP-BM could participate in the modifications of gut–brain axis activity controlling glycemia. This action could be of major importance since we do not observe any effect on insulin release in all groups suggesting that the potential action of GLP-1 is independent of the well-described incretin effect of this hormone. Reinforcing this hypothesis is the fact that first, intestinal GLP-1 can stimulate the release of NO from nNOS neurons in the ileum in order to communicate with the brain via afferent nerves [20] and second, that GLP-1 could be now considered as enterosynes [21].

Several questions remain to be resolved with future experiments. First, could the effects of HHP-BM observed in our study be due to apelin alone or GLP-1 alone or in combination with other enterosynes? This point could be raised by assessing the levels of other molecules (peptides, lipids) present in HHP-BM that could act on enteric neurons or other cellular targets in the digestive tract or in other organs after intestinal absorption. Second, which factors are implicated in the increase in *nNos* mRNA level in the ileum? GLP-1 is known to increase the activity of nNOS in myenteric neurons leading to NO release that inhibits the mechanical activity of the intestine [22]. Whether milk GLP-1 or other milk factors can influence the expression of nNOS in the intestine remains to be determined. Another mode of action of GLP-1 that could explain the decrease in ileum contraction in HHP-BM treated mice is the fact that GLP-1 receptors are also expressed on intestinal smooth muscle cells since the agonist GLP-1 receptor, i.e., exendin-4 has an inhibitory action on colonic motility in rats [23]. Finally, the experimental model used in this study is questionable has it has been performed in adult male mice, but it represents a preliminary study that allowed us to study the effect of BM treatments on several intestinal segments ex vivo and to evaluate in vivo the intestinal gut–brain axis implication in glucose homeostasis after an oral glucose tolerance test. Future studies using newborn mice will allow us to evaluate other digestive effects of these treatments of BM on, for example, the integrity of the intestinal barrier (i.e., mucus production, permeability, gut immunity), the maturation of the gut mucosa and the establishment of the gut microbiota.

## 5. Conclusions

To conclude, we have discovered that HHP protocol preserves both apelin and GLP-1 levels in human milk, demonstrating the potential clinical importance of this original sterilization method. Based on our observations, we postulate that HHP-treatment of BM could improve glucose homeostasis and the gut–brain axis activity in preterm newborns.

## Figures and Tables

**Table 1:** Primers sequences.

Gene of Interest	Forward Sequence	Reverse Sequence
*Hprt*	GTTCTTTGCTGACCTGCTGGAT	CCCCGTTGACTGATCATTACAG
*nNos*	ACGTCAAGTACGCCACCAACA	GCGAGTTCCACACTCGGAAGT
*Chat*	TGATCTTTGCTCGGCAGCACT	TTGGCCCAGTCAGTGGGAATG

## Data Availability

Data of this study are available upon request to authors.
